# A Wellness Mobile Application for Smart Health: Pilot Study Design and Results

**DOI:** 10.3390/s17030611

**Published:** 2017-03-17

**Authors:** Giovanna Sannino, Manolo Forastiere, Giuseppe De Pietro

**Affiliations:** 1 Institute of High Performance Computing and Networking (ICAR) of National Research Council (CNR), 80131 Naples, Italy; giuseppe.depietro@icar.cnr.it; 2 NEATEC S.p.A., Pozzuoli, 80078 Naples, Italy; m.forastiere@neatec.it

**Keywords:** Wellness App, lifestyle monitoring, pilot study

## Abstract

Wellness is one of the main factors crucial in the avoidance of illness or disease. Experience has shown that healthy lifestyle programs are an important strategy to prevent the major shared risk factors for many diseases including cardiovascular diseases, strokes, diabetes, obesity, and hypertension. Within the ambit of the Smart Health 2.0 project, a Wellness App has been developed which has the aim of providing people with something similar to a personal trainer. This Wellness App is able to gather information about the subject, to classify her/him by evaluating some of her/his specific characteristics (physical parameters and lifestyle) and to make personal recommendations to enhance her/his well-being. The application can also give feedback on the effectiveness of the specified characteristics by monitoring their evolution over time, and can provide a positive incentive to stimulate the subject to achieve her/his wellness goals. In this paper, we present a pilot study conducted in Calabria, a region of Italy, aimed at an evaluation of the validity, usability, and navigability of the app, and of people’s level of satisfaction with it. The preliminary results show an average score of 77.16 for usability and of 76.87 for navigability, with an improvement of the Wellness Index with a significance average of 95% and of the Mediterranean Adequacy Index with a significance average of as high as 99%.

## 1. Introduction

Wellness and lifestyle are key determinants of, on the one hand, good health, and, on the other, of disease, disability and even premature mortality. The modern urban lifestyle is characterized by insufficient physical activity, junk food, and high stress levels, all of which affect people’s well-being. In the long term, this kind of lifestyle can lead to health problems and diseases, such as excessive weight, obesity, strokes, cardiovascular diseases, and diabetes [1].

Well-being, fitness, and the state of health in general, promoted through proper exercise, diet, and sleep habits, are priorities in many countries [2]. According to the World Health Organization, approximately 31% of adults are insufficiently active, and approximately 3.2 million deaths each year are due to insufficient physical activity [3]. Additionally, study [4] reports that 86% of deaths, 77% of the loss of years of healthy life and 75% of healthcare costs in Europe are caused by certain diseases (cardiovascular diseases, cancers, diabetes mellitus, chronic respiratory diseases, mental health problems and muscular-skeletal disorders) that have in common *modifiable risk factors*, such as smoking tobacco, excessive weight and obesity, alcohol abuse, the low consumption of fruit and vegetables, physical inactivity, excess fat in the blood and high blood pressure. These risk factors, alone, are responsible for 60% of the loss of years of healthy life in Europe.

The examination of behavioral risk factors, the evidence of their causal relationship with disease, and their prevalence in the population, together with the combined effects of such factors, have led researchers in medicine to the conclusion that correcting a few basic behaviors and lifestyle problems could have a major positive effect on the health of the population [4].

On the other hand, it is not easy to change the lifestyles of people, which are based on habits, preferences and tastes, reinforced by organizational needs and consolidated over time. Despite the widespread dissemination of information, characteristic of the modern era, and the major publicity campaigns on specific issues such as the dangers to health of certain behaviors (such as smoking or drinking alcohol), the majority of the population do not adopt a lifestyle adequate to protect their health.

ICT technologies and, in particular, domain-specific Wireless Sensor Networks named Wireless Body Sensor Networks (BSNs) have enormous potential to positively affect the daily life of people [5,6] and to help people to change their unhealthy behaviors. A BSN is constituted of a set of wearable sensors that communicate with a local and/or personal coordinator device, such as a smart phone or tablet, to provide a real-time, continuous and non-invasive monitoring of assisted living.

A key benefit of this technology is the possibility of continuously monitoring vital and physiological signs without obstructing the comfort of the user when performing her/his daily activities. Indeed, in the last few years, the diffusion of BSNs has increased enormously with the introduction, at a mass production level, of smart wearable devices (particularly smart watches and bracelets) that are able to capture certain parameters such as body acceleration, electrocardiogram (ECG), pulse rate, and bio-impedance readings [7,8].

Within the ambit of the Smart Health 2.0 project [9], we have developed a WellnessApp [10], based on the assumption that people need to follow consistently a certain general course of action, which includes recommendations and suggestions that are suited to the individual’s specific case. A “personal trainer”, as they say, is not surprisingly a specialist much in demand today. The ambition of this app is to provide people with something similar, namely an application that, after collecting a bank of information on the subject by means of a BSN, is able to “classify” her/him based on her/his individual characteristics (physical parameters and lifestyle) and then to propose specific recommendations to improve her/his well-being. By monitoring the evolution over time of these individual characteristics, the application can also give feedback on the effectiveness of the measures adopted and therefore provide a positive stimulus to motivate the subject to continue the path that she/he has taken.

In order to evaluate the validity and the usability of the developed app, and people’s level of satisfaction with it, we have drawn up an appropriate trial protocol, a detailed document in which we have described the complete execution plan, focusing on organizational and ethical aspects. We have analyzed several studies existing in literature, for example [11,12,13] and, based on this analysis, we have built a trial protocol in accordance with the SPIRIT 2013 Statement [14], which provides guidelines on the performance of a clinical study.

The remaining sections of the paper are organized as follows: in Section 2, we present a literature review of the health and fitness mobile applications currently available on the market; in Section 3, we report details of the Wellness App developed, while in Section 4, we present the pilot study, describing initially the methodology used in its design, and, secondly, specific information relating to the evaluation of the app; in Section 5, we present the trial protocol execution, and in Section 6, we discuss the results of the study; finally, in Section 7, we present some conclusions.

## 2. Literature Review

Nowadays, app markets provide people with an ever increasing number of applications, about 40,000 of which are related to healthcare, namely “health and fitness” apps or mHealth Apps [15]. Of these, 23,490 are available on Apple iTunes (for iOS-based devices) and 17,756 on Google Play (for Android-based devices).

These apps are widely used in medical practice in several fields including chronic disease, such as, for example, the follow-up of people with diabetes mellitus, or the monitoring of weight loss in obesity subjects. Most of the popular apps for healthcare and fitness aim providing people with services to cover a wide range of functionalities, including self-monitoring, relating to, for example, the improvement of fitness activity, the measurement of health and fitness parameters, determination of the Body Mass Index (BMI), the setting of weight goals, advice on nutrition and diet, and the monitoring of sleep. More generally, the apps involve the setting of new health and fitness targets, and also provide assistance in the achievement of these goals by promoting changes in behavior.

Additionally, several initiatives have been undertaken aimed at safeguarding and improving long-term health and promoting healthy aging. Researchers and developers are strongly involved in realizing wellness applications [16,17,18], ranging from heart-rate activity monitoring, and step counters, to applications for the tracking of eating habits and physical activity.

Simultaneously with the development of these mHealth Apps, the number of studies providing reviews of their effectiveness has also been increasing, with particular reference to misleading content, the lack of an evidence base, and the absence of any medical professional involvement in the app design.

A very interesting study [19] examines several apps selected from the best rated apps downloaded from iTunes and Google Play. The results reveal important evidence about the current limitations of these apps, summarized as follows:

- the lack of rigorous evidence-based research trials whose results are peer reviewed by independent experts to determine whether the app is safe and effective;
- the lack of long-term and large sample size trials with heterogeneous participants in terms of age, gender, socio-economic status, personally type, and smartphone and social networking experience to assess the safety and efficacy of the app;
- the lack of emphasis on evidence-based and medical professional involvement;
- the requirement that a smartphone is always available during the performance of the activity;
- the lack of evidence-based strategies or established behavior change theories as the basis of the app development;
- the use of smartphone sensors instead of stand-alone sensor devices which have been demonstrated to be more accurate [20];
- the lack of any interoperability with other health and fitness apps and devices;
- the lack of any behavior change techniques integrated into the app (e.g., YouTube videos or Tweets);
- the lack of a user-friendly layout for an easy adoption and use of such technology.

Taking into account these considerations, we have designed and developed a new Wellness App, with the aim of addressing these issues, in that it has a documented evidence base, includes the incorporation of several behavior change techniques (BCTs) [21], and involves a high degree of medical professional involvement. According to the taxonomy described in [21] that identified 26 behavior change techniques, the interesting study of Middelweerd et al. [22] reviews the most well known apps developed for iOS and Android that promote and monitor physical activity and/or a healthy diet. Table 1 shows the behavior change techniques (BCT) score obtained by the proposed Wellness App in comparison with the apps reported in study [22].

Unfortunately, this study does not report the list of the BCTs for each app, but only the total BCT score, which is the sum of the BCTs included in each app. For this reason, it is not possible to make a detailed comparison. However, it is important to remark that our proposed Wellness App includes 9 behavior change techniques, one more than the app with the best score reported in study [22], namely the “RunKeeper App”.

The following is a list of the BCTs included in the proposed Wellness App:

(i) **Providing information about the behavior-health link**: the app contains a section with information and literature references relating to the correct habits useful for a healthy lifestyle for the prevention of chronic diseases.
(ii) **Prompting intention formation**: the Wellness App suggests and encourages the user to take actions to support a resolution aimed at changing behavior, such as taking more exercise in the following week.
(iii) **Setting graded tasks**: the app, by using the reasoning module, plans what the user should do by defining specific behavior changes scheduled with the timing and different effort levels required.
(iv) **Providing instruction**: telling the user how to perform an activity and/or preparatory activity.
(v) **Prompting specific goal setting**: this is directed towards encouraging the user to decide to change a behavior or maintain a behavioral change. This is provided by means of a reasoning module that analyzes the wellness performance indicators and decides which kind of suggestions are necessary to direct the user towards the adoption of correct behaviors. For example, the app could suggest an increase in the time devoted to physical activity if the Time spent per Week on Physical Activity Index is too low in comparison with WHO guidelines.
(vi) **Prompting the self-monitoring of behavior**: the user is asked to keep a record of specified behavior/s as a method for changing behavior. This facility is guaranteed by providing the app with mechanisms to monitor performance and store all monitored information.
(vii) **Providing feedback on performance**: the app calculates different performance indicators relating to the monitored user’s wellness and lifestyle data. For example, the proposed Wellness App is able to calculate the Mediterranean Adequacy Index that indicates how close the user’s diet is to the Mediterranean diet that is considered ideal by many researchers and doctors.
(viii) **Providing contingent rewards**: this involves using praise or rewards for attempts at achieving a behavioral goal. Every time the app detects a good performance index value, it sends a congratulation message to the user in order to reward the performance of a correct behavior. In this way, the app provides a motivational support for the user, so reducing the dropout rate.
(ix) **Time management**: the app is provided with a mechanism that reminds the user, at specific times, to perform some form of physical activity.

## 3. The Wellness App

Different parameters are recorded daily by the proposed wellness mobile application using several mechanisms, both automatic and manual. Appropriate indicators that measure the type of nutrition, the amount of physical activity and the sleep quality are calculated and monitored by the proposed Wellness App in order to encourage the user to improve her/his lifestyle. These indicators are analyzed by an inference reasoning module that decides which kinds of suggestions the subject needs in order to be directed towards the adoption of correct behaviors, such as for example the correct maintenance of the Mediterranean-type diet, that is recognized as the best diet for the prevention of cardiovascular disease, and/or the improvement of the physical activity plan.

**Figure 1:**
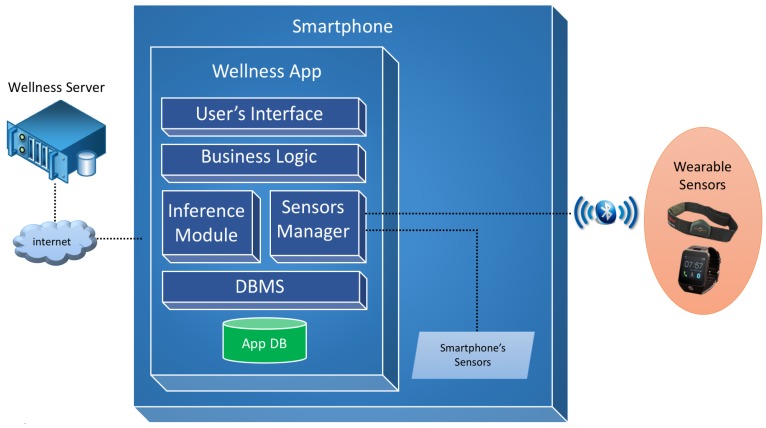
Overview of the software architecture.

The design of the software architecture of the proposed Wellness mobile application is shown in Figure 1.

The **User’s Interface module** is a software layer that contains all the interaction interfaces provided to the user and the mapping phase of these interfaces with the business layer, thereby realizing the Model View Controller (MVC) design pattern.

The **Business Logic module** provides all the logic required to manage the diet diary, physical activity diary, user profile and privacy logic and the calculation of the lifestyle indexes that are described in Section 6.

The **Inference module** manages the cycle of reasoning and acts as a main interface between the system of reasoning and the business module. Through this module functions are implemented to provide indications to the user with regard to all the monitored parameters and inputs.

The **Sensor Manager module** deals with the dialogue between the app and the available sensors by creating a virtual layer and allowing the application with different sensors. It is responsible for managing the data exchange via the Bluetooth communication protocol with sensors, such as wristbands, smart watches or smart elasticized chest-belts, like for example the Zephyr BioHarness [23].

The **DBMS module** manages the database and the persistence of the Wellness data. Moreover, it also provides mechanisms for the exchange of information between the app and a **Wellness Server** [24], through the use of apposite REST services, for data synchronization, historic data recovery and the updating of the user’s goals.

The software has been realized in order to provide the best performance to the user and to be maintainable, dependable and usable. To achieve this objective, we have followed software engineering principles, starting from the elicitation, analysis, and specification of requirements, summarized in a formal document according to standard guidelines [25].

**Figure 2:**
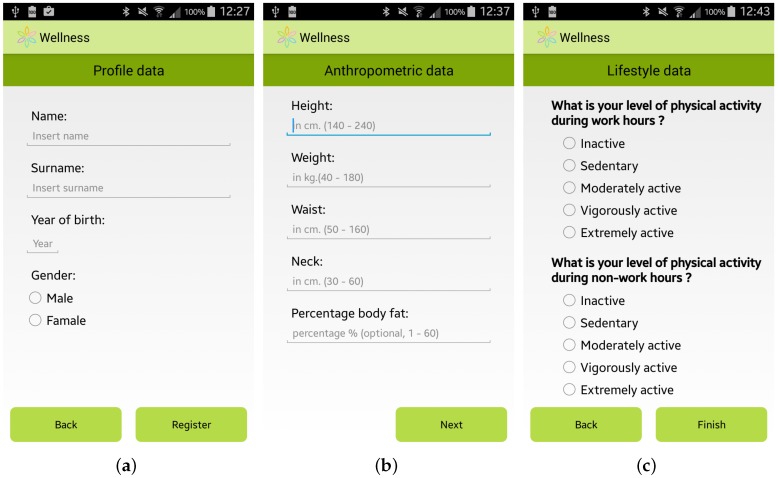
Screenshots of the Wellness App: (**a**) General Information; (**b**) Anthropometric Information; and (**c**) Lifestyle Information.

The expert team involved in the SmartHealth 2.0 project, which is composed of specialists from the Mediterranean Diet Foundation and from the University “Magna Graecia” of Catanzaro (Italy), identified a methodology to study the habits and lifestyle behaviors of a user. This user assessment is automatically performed by the app by providing the user with a questionnaire to elicit general information, including her/his anthropometric parameters, profession and habits relating to physical activity, nutrition, smoking, and alcohol consumption. Some screenshots of the questionnaire for the user assessment are shown in Figure 2.

The data requested for the user assessment are also used by the app to personalize the suggestions to improve the well-being of each specific user. It should be noted that some of the requested information is not mandatory, but is automatically calculated by the Wellness App by using basic mandatory data, like for example the Body Fat Percentage which is automatically estimated by using the measurements of the height and the weight of the user [26].

**Figure 3:**
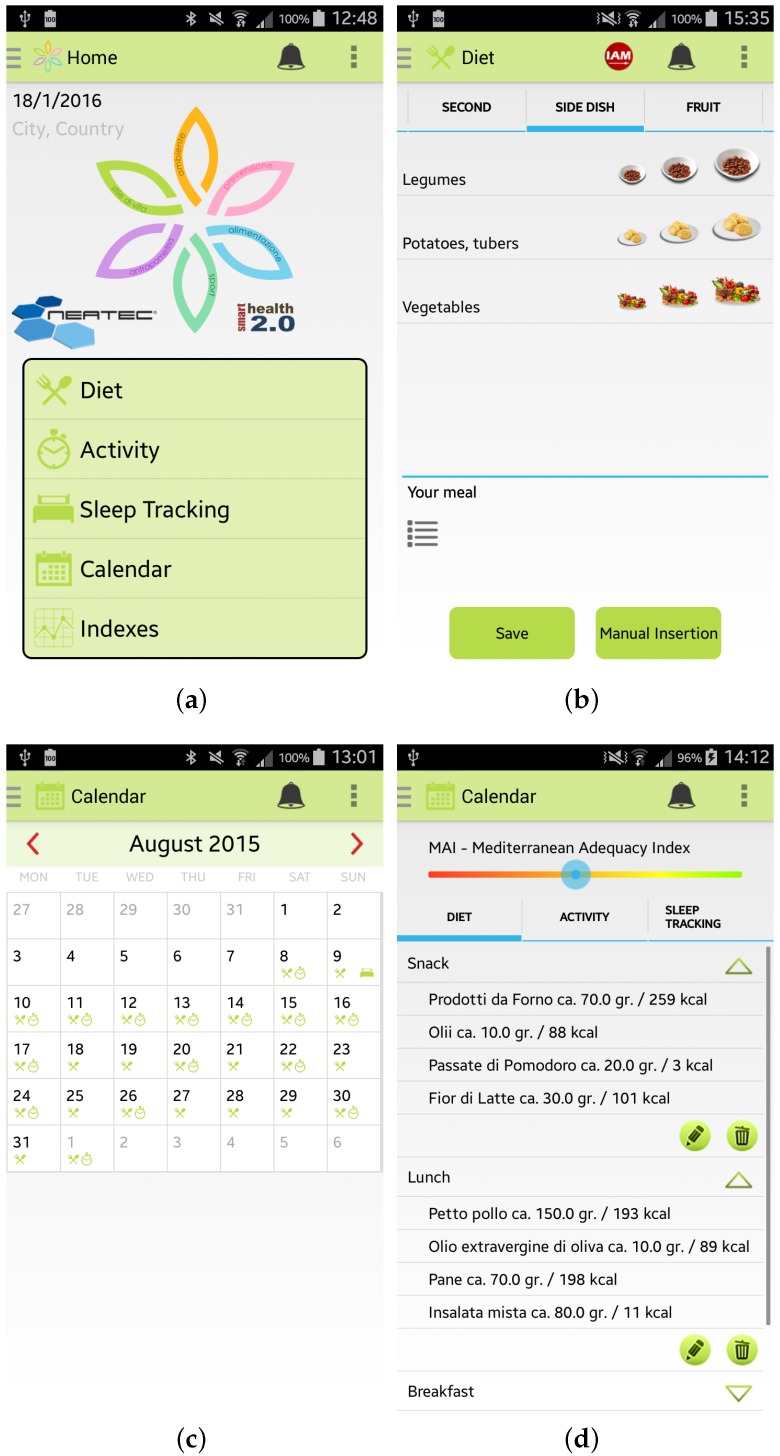
Screenshots of the Wellness App: (**a**) Main Menu; (**b**) Insertion of a new meal; (**c**) Calendar; and (**d**) Details.

Once the user has inserted her/his information, she/he can access the main menu of the Wellness App. From the main menu, shown in Figure 3a, by selecting, for example, the specific option “Diet” the user can insert information about a new meal. The app provides the user with a simple layout to facilitate the interaction during the insertion of new data. For example, some pre-set quantities have been provided to speed up the insertion of new meal data, as shown in Figure 3b. Some examples of which kind of food can be inserted in the Wellness App as meals are reported in [10]. Every day and every week the Wellness App automatically calculates the Mediterranean Adequacy Index (MAI) based on the inserted meals. The MAI index assesses how close the diet is to the Reference Mediterranean Dietary Pattern. This index is obtained as illustrated in study [27].

The user can visualize all the inserted information by selecting the Calendar button from the main menu. As shown in Figure 3c,d, the user can have a monthly view, or a daily view where she/he can check, modify or delete the added data.

**Figure 4:**
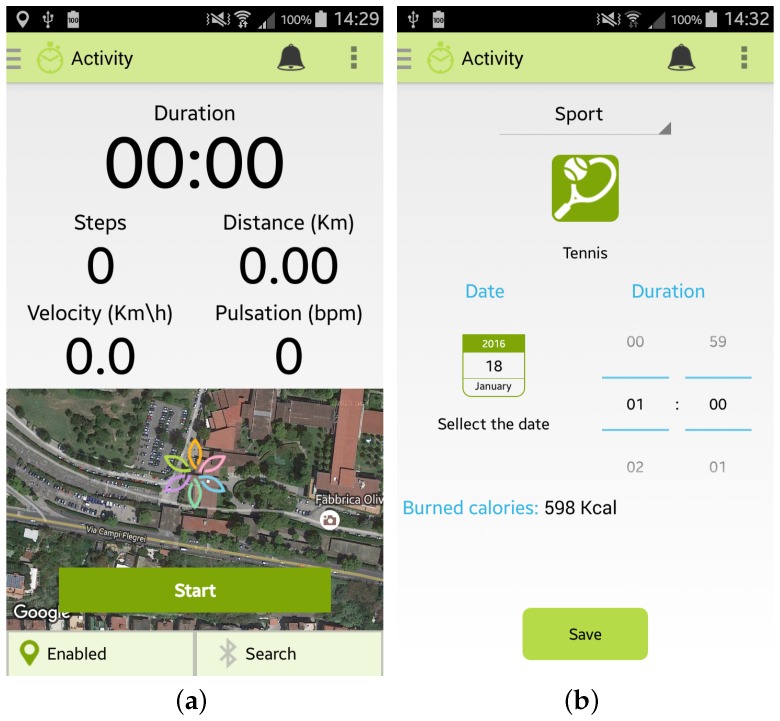
Screenshots of the Wellness App relating to physical activity: (**a**) Automatic insertion of a new activity; (**b**) Manual insertion of a new activity.

Furthermore, the user can monitor her/his Physical Activity by selecting from the main menu the specific option “Activity”. Two modalities are provided to the user by the Wellness App:

- the “automatic monitoring” of activities that uses the inertial sensors of the smartphone or an external sensor, such as a smart band, connected to the app by a Bluetooth connection. As shown in Figure 4a, the app is able to monitor the number of steps performed, the distance travelled (km), the velocity (Km/h) and the heart rate (bpm) if provided by the sensor. To use this option it is necessary to carry both the smartphone and the sensor during the activity;
- the “manual monitoring” of activities that permits the user to manually select the kind of activity performed, the duration, and the date, as shown in Figure 4b. This option has been devised in order to give the user the possibility of freely performing the activity, without the necessity of carrying her/his smartphone, and then inserting the type and the amount of the activity performed. Some examples of which kinds of activity can be manually inserted in the Wellness App are reported in [10].

**Figure 5:**
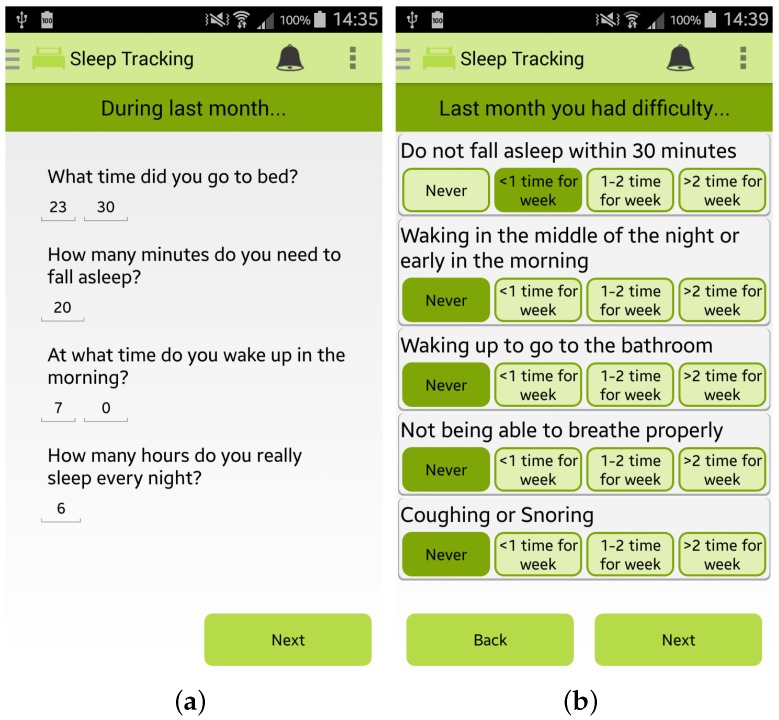
Screenshots of the Wellness App relating to sleep monitoring: (**a**,**b**) PSQI questionnaire for sleep quality estimation.

Finally, the app permits the user to estimate her/his sleep quality by completing the PSQI questionnaire, one of the most popular tools for assessing the quality of sleep [28], as shown in Figure 5a,b.

**Figure 6:**
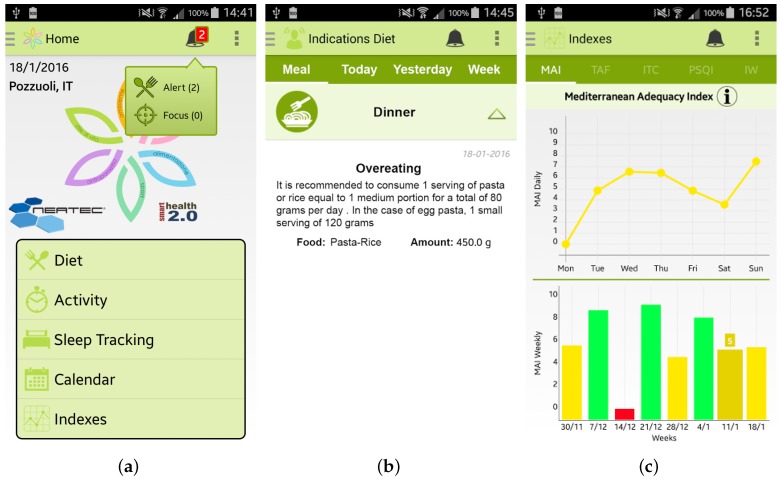
Screenshots of the Wellness App relating to the alerts and the statistics of the calculated indexes: (**a**) the main menu of the app where an alert is notified; (**b**) detailed view of the alert; and (**c**) daily and weekly trends of the Mediterranean Adequacy Index (MAI).

Every day, or every time the user inserts any new data, the app automatically estimate different indicators, such as the Mediterranean Adequacy Index. Every time the app detects an anomalous situation, it promptly alerts the user, as shown in Figure 6a,b. By clicking on the alert, the user can visualize the personalized recommendations to follow to improve her/his wellness and lifestyle. It is important to note that all the recommendations provided by the app are based on expert knowledge formalized in close collaboration with the medical team of the University of Magna Graecia of Catanzaro (Italy) involved in the SmartHealth 2.0 project. Finally, all the calculated indexes are available to view by selecting the Index button from the main menu of the app, such as, for example, the MAI index in Figure 6c.

## 4. The Pilot Study Design

The experimental protocol consists of several sections:

- Administrative: where the “Title” and an identification of the study and/or trial, the roles and the responsibilities of the staff involved in the study and details of any financial support are described.
- Introduction: in this section the main objectives of the study, including a set of the relevant and related published studies, the outcomes, both primary and secondary, of the trial process, and the interpretation of the results are described.
- Methods: the protocol also provides the major design elements of the study and/or trial, such as, for example, the phases of the trial and the randomization scheme. Additionally, a description of the study, including the number of the study groups, the size of each study group, if applicable, and the duration and sequence of the trial periods is here detailed.
- Ethics and Dissemination: in this section the ethical approval and any rules and behavioral norms of the participants are specified.
- Appendix: the protocol, if necessary, also provides a section containing documents useful for the trial process, such as the informed consent, the information sheet, and the test for the usability evaluation.

Some details of the pilot study designed for the wellness application are reported in Table 2.

## 5. Pilot Study Execution

The trial was conducted in four urban areas in the Calabria region, namely Cosenza, Crotone, San Giovanni in Fiore, and Lamezia Terme. As remarked in the Methods section of the trial protocol reported in Table 2, we divided the study into three phases. We started with the participant recruitment phase with the promotion of the testing program through information campaigns as documented in [29,30,31], by means of posters and brochures, and news reports published on the project web-site [9] and on the main social networks, such as Linked-In and Facebook.

The total number of people recruited was 40, aged between 18 and 65 years old. Table 3 shows the number of individuals enrolled in each urban area, the effective number of Wellness apps installed, and the number of anamnestic forms (Sheets), containing the medical history of the participant, and usability questionnaires collected.

**Figure 7:**
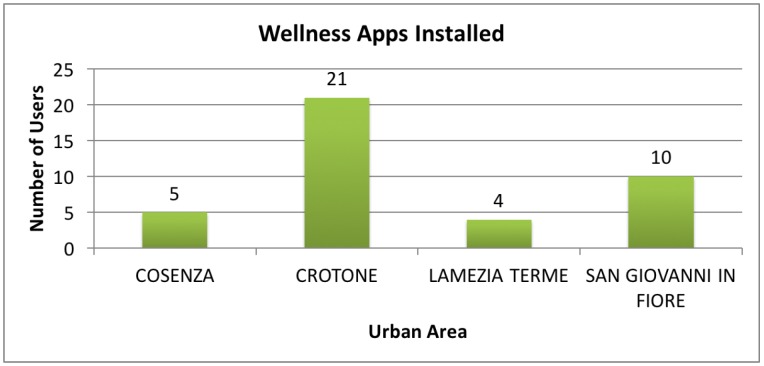
Details about the deployment of the Wellness App.

In Figure 7 it is possible to see a graphical representation of the distribution of the installed apps between the different urban areas.

For each person who expressed an interest in participating in the trial, possession of the eligible criteria requirements was verified. If this was confirmed, the individual was able to access the second phase of the trial. Here, we provided information on the scope of the study and the procedures of the trial process and informed the participants that it was possible to abandon the experimental practice whenever they wanted.

The interested individuals were then registered for the trial by signing the anamnestic form with all their personal, medical and contact information, a form for confirmation of the informed consent, and the privacy statement essential to guarantee data protection.

The trial process then proceeded with three meetings with the participants distributed over two months. During the first meeting we installed the Wellness App on their mobile devices and explained its functionalities. It is important to note that the Android operating system is required for the installation and use of the app. Therefore, for each participant who did not have an Android-based smartphone, we provided a Mediacomm Phonepad G501 smartphone on loan for use throughout the duration of the study.

In the second meeting, in order to increase involvement in the trial process, the participants were updated on the progress of the project, as well as provided with a new improved version of the Wellness App realized in accordance with the indications suggested by the participants during the previous meeting. In the third and last meeting we collected all the data from the mobile devices of the participants, and talked with each of them, encouraging them to discuss the use of the app, any difficulties encountered during the trial, the perceived quality of the information provided, and any preferences for different features. In this latter meeting we also distributed the usability test questionnaires, requesting the participants to complete them, providing spontaneous responses. All the questionnaires were anonymous.

## 6. Pilot Study Results

Out of a total of 40 initial Wellness App installations, only 24 people were considered to have effectively used the app. For each of these individuals, the average results of the initial and final values, with reference to the following four different indicators, are shown:

1. the **MAI index**, the “Mediterranean Adequacy Index” [32], which measures the type of diet in relation to the Mediterranean diet, considered ideal by many researchers;
2. the **TCI index**, the “Target Calorie Index” [33], which measures the amount of calories consumed in relation to the optimal value for the individual calculated in accordance with her/his anthropometric data;
3. the **TPA index**, the “Time spent per week on Physical Activity Index” [34], which measures the amount of physical activity in comparison with the WHO guidelines; and
4. the **PSQI index**, the“Pittsburgh Sleep Quality Index” [28], which estimates the quality of sleep.

Based on these indicators, the Wellness App calculates also a new index, the Wellness Index (WI), which provides a measurement, albeit partial and incomplete, of the state of physical well-being of the subject.

This WI index is calculated once a week and depends, as shown in Equation (1), on the mean values of the MAI index ($\bar{MAI}$), TPA index ($\bar{TPA}$), TCI index ($\bar{TCI}$), and PSQI index ($\bar{PSQI}$) during that weekly period.

(1) $$WI=\frac{\bar{MAI}\times 2+\bar{TPA}\times 2+\bar{TCI}+\bar{PSQI}}{6}$$

All the collected data were evaluated by performing a filtration, thus eliminating incomplete data, and by establishing the proportion of the significance index, in relation to the initial and final data.

**Figure 8:**
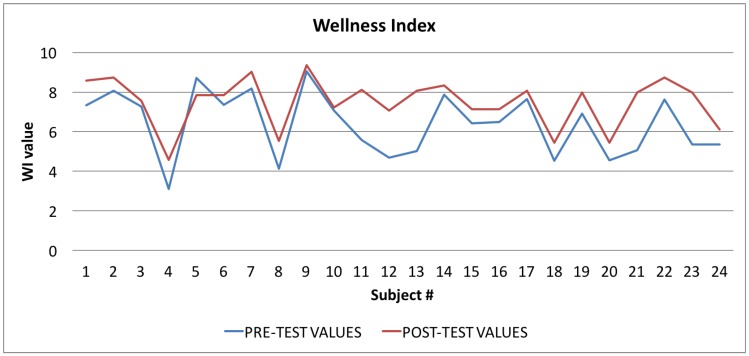
Wellness Index: averages of the values calculated at the beginning (blue line) and at the end of the trial (red line).

**Figure 9:**
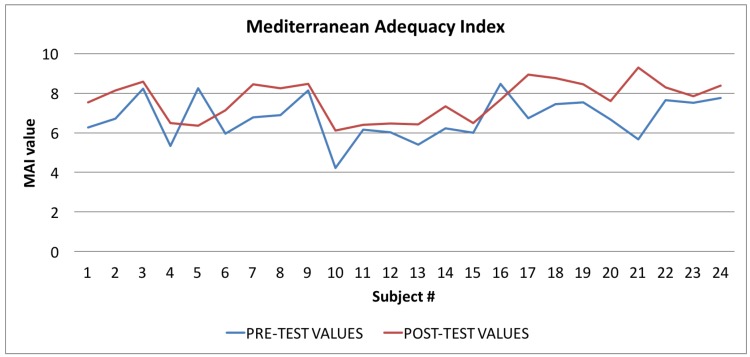
Mediterranean Adequacy Index: averages of the values calculated at the beginning (blue line) and at the end of the trial (red line).

**Figure 10:**
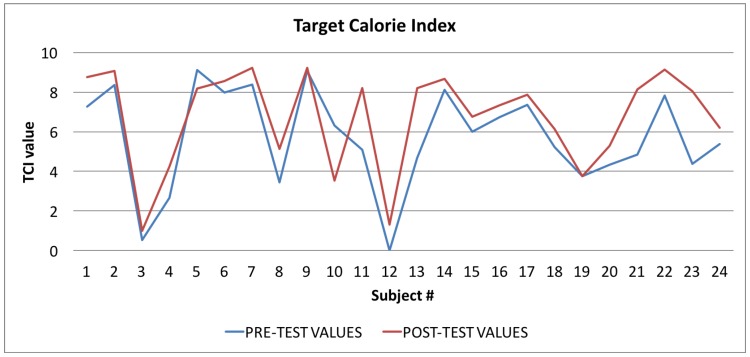
Target Calorie Index: averages of the values calculated at the beginning (blue line) and at the end of the trial (red line).

In Table 4 all the values of the indexes calculated by the Wellness App at the beginning of the study and the same indexes calculated by the app at the end of the study are reported. To evaluate the goodness of the app, we carried out a comparison for each index between the averages of the values for each index for the sample of 24 individuals. As shown in Table 4, and then in a more easily readable way in Figure 8, Figure 9 and Figure 10 and Figure 12 for each index we obtained an improvement.

In Figure 8 the graph showing the average values of the Wellness Index (WI) is reported: the values calculated at the beginning of the trial phase are in blue, and the values calculated at the end of the trial phase are in red. The figure shows that, in the vast majority of cases (96%), there was an improvement in the value of the Wellness Index.

In Figure 9 the graph showing the average values of the Mediterranean Adequacy Index (MAI) is reported: the values calculated at the beginning of the trial phase are shown in blue and the values calculated at the end of the trial phase are shown in red. As for the WI index, the figure shows that, in most cases (92%), there was an improvement in the Mediterranean Adequacy Index.

**Figure 11:**
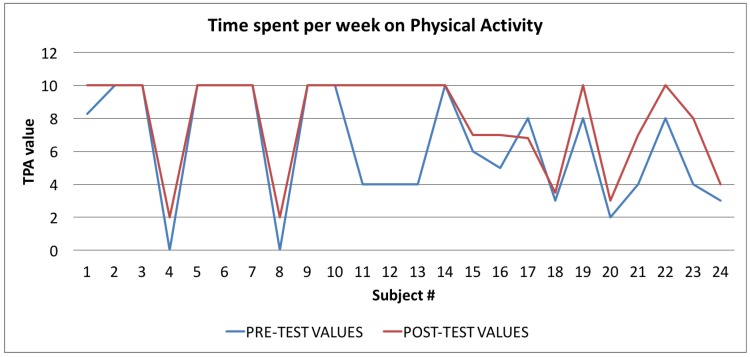
Time spent per week on Physical Activity Index: averages of the values calculated at the beginning (blue line) and at the end of the trial (red line).

**Figure 12:**
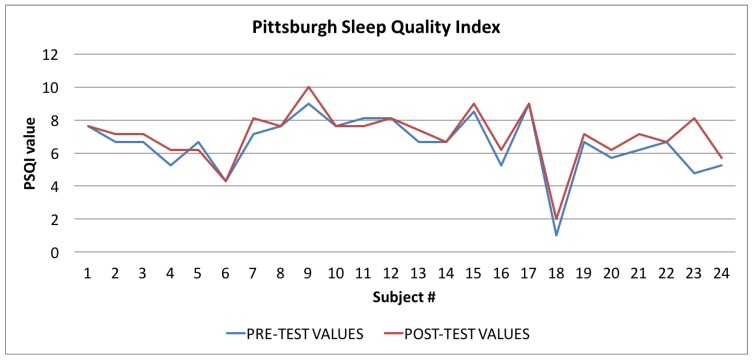
Pittsburgh Sleep Quality Index: averages of the values calculated at the beginning (blue line) and at the end of the trial (red line).

Finally, in Figure 10, Figure 11 and Figure 12, the average values of the Target Calorie Index (TCI), the Time spent per week on Physical Activity Index (TPA), and the Pittsburgh Sleep Quality Index (PSQI) are reported, respectively. The values calculated at the beginning of the trial phase are shown in blue and the values calculated at the end of the trial phase are shown in red. As for the WI and MAI, the figures show that there were improvements in each case, of 91.6%, 95.8%, and 91.6%, respectively.

### 6.1. Index Evaluation: Statistical Analysis

We used the “*t*-Student Test” Test [35] (2) (for unpaired data), to verify whether the differences between the averages are attributable to the null hypothesis.

(2) $$t=\frac{\bar{X_{i}}-\bar{X_{j}}}{\sqrt{\frac{s_{i}^{2}\left(n_{i}-1\right)+s_{j}^{2}\left(n_{j}-1\right)}{n_{i}+n_{j}-2}\left(\frac{1}{n_{i}}+\frac{1}{n_{j}}\right)}}$$

where $\bar{X_{i}}$ and $\bar{X_{j}}$ are the averages of the two groups (the initial and final values of each index), $s_{i}^{2}$ and $s_{j}^{2}$ are the variances of the two groups, and $n_{i}$ and $n_{j}$ are the numerosities of each group.

The null hypothesis ($H_{0}:\mu_{1}=\mu_{2}$) is that there is no difference between the groups with respect to the considered parameter. So if the null hypothesis is valid, any differences observed in the samples (the considered parameter) can be attributed to chance alone.

Using the *t*-Student test we calculated the probability that the null hypothesis is not true (that is, the sample means that $\bar{X_{i}}$ and $\bar{X_{j}}$ are different not only on account of random factors due to the extraction of the sample).

The admissible level of significance, α, is equal to 0.05 (5%). In contrast, the test is considered significant if the probability associated with the null hypothesis is less than 5%.

**Figure 13:**
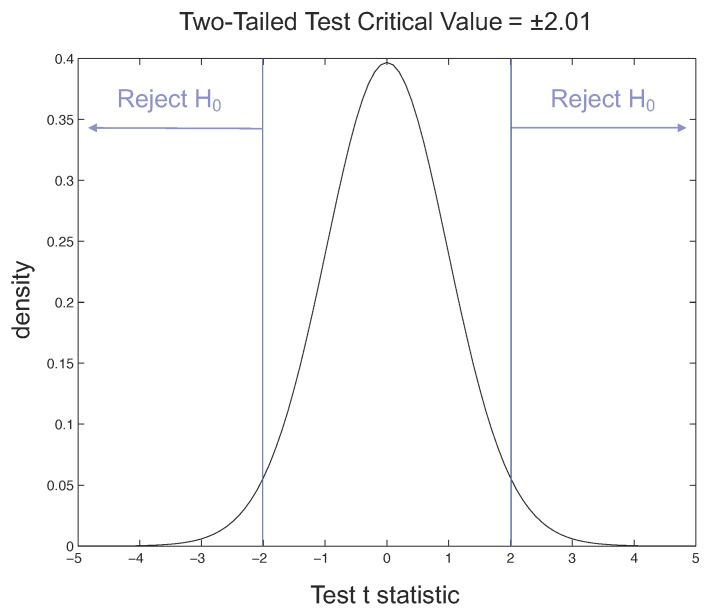
Rejection regions for two-tailed *t*-test with α = 0.05.

Considering the *t*-Student distribution, if we have α=0.05 and a value of the degrees of freedom equal to 46, for our two-tailed *t*-test the critical value is 2.01. The rejection regions are shown in Figure 13, where the probability density function of the normal distribution, also called the Gaussian curve, is drawn and the tail, where observations lead to the rejection of the null hypothesis, is indicated.

By applying Equation (2) to the samples related to the Wellness Index (WI), shown in Table 4, we obtained a *t* value of −2.5267, and a *p*-value of 0.015. The returned value of H = 0 indicates that the *t*-Student test rejects the null hypothesis at the 5% significance level.

The *t*-Student test indicates that the difference between the observed averages is significant by more than 95%, and therefore the difference between the values calculated before and after the test is almost certainly due to the “treatment” (more than 98% of probability) and is not random.

For the Mediterranean Adequacy Index (MAI), the *t*-Student test results indicate that the difference between the averages observed is significant, from 95% to 99%, with a *p*-value of 0.0035. In fact, by applying Equation (2) to the MAI index, we obtained a t value of −3.0820. In this case also, the returned value of H = 0 indicates that the *t*-Student test rejects the null hypothesis at the 5% significance level.

Very significant results are also obtained for the TPA index (92% probability in favor of the treatment), while slightly lower values are obtained for the TCI and PSQI indexes (85% and 68.5% significance, respectively).

It is interesting to note that the WI index is both the most significant because it is practically “measured live” and also the most challenging in terms of the graphical use of the Wellness App. On the contrary, the PSQI is calculated from a questionnaire with quite subjective responses, that typically require a certain time interval before changing, even in terms of the way in which they are expressed. It would have been interesting to “measure” in a timely manner the quality of sleep by means of the sensors, but it would have been necessary to wear these every night and, in any case, the data that would have been obtained would not have been easy to interpret.

The overall feeling derived, however, in line with expectations, however, is that the effectiveness of the Wellness App in improving an indicator of well-being also depends greatly on the graphics and the ease of use associated with it, as well as the interest the specific user has in that specific indicator. This is also why, in the last stage of the trial, questionnaires were administered to measure the usability of the app and subsequently to improve it in accordance with the feedback received.

### 6.2. Usability Results

During the final meeting, the participants were asked to complete, very spontaneously, the usability test provided in the trial protocol. The aim of the usability questionnaires was to obtain feedback on the real result of the interaction with the app, by measuring the speed and effectiveness of the experience of use. The test consisted of 20 questions: the first 10 questions were from the SUS questionnaire (System Usability Scale) [36], a test used in many studies of usability, proven to be a reliable tool of comprehensive assessment.

The remaining questions were aimed at evaluating the effective understanding, navigability, and facility of the user interfaces provided by the Wellness App [37].

To ensure a correct consideration of the SUS result, the questionnaire was completed only after the user had had a chance to use the app during the trial study. Each participant was asked to answer the questions in an immediate way, without pausing for a longer consideration. The test produced a single number that represented a composite measure of the overall usability of the app. The test scores varied in the range of 0–100.

**Figure 14:**
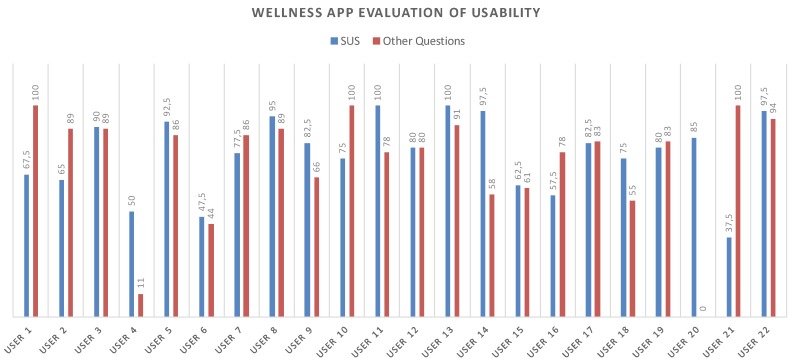
SUS and Navigability scores related to each participant.

The tests results showed fairly positive feedback, confirming the positive aspects of the app and emphasizing its upgrading. In this regard, the test also included an open-ended question to collect any comments from the users. Figure 14 shows all of the obtained values for each test. The average value for the SUS questionnaire is 77.16 while, instead, for the other questions the navigability average score is 76.87.

**Figure 15:**
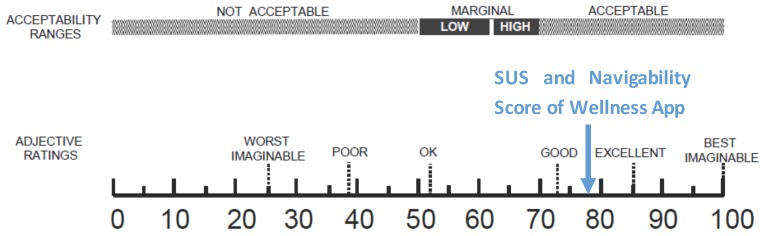
A comparison of the adjective ratings, acceptability scores, and school grading scales, in relation to the average SUS and Navigability scores.

The number of collected questionnaires was lower than the number of people involved in the trial study (22 questionnaires out of a total of 40 installed apps) due to the poor turnout at the last meeting. Starting from the collected data concerning the SUS, the level of usability and acceptability of the app [38] could be evaluated by means of the qualitative adjective [37]. As shown in Figure 15, both the SUS and the Navigability scores obtained from the evaluation of the Wellness App are collocated between *Good* and *Excellent* values.

Finally, we want to emphasize that, even if the usability and navigability tests were completely anonymous, we did not receive any feedback with a particularly low score. However, according to the indications received in the field, it is assumed that, in the majority of cases, some difficulties were generated in terms of the use of this new technology by participants over 60 years of age.

## 7. Conclusions

The statistical tests performed on the data collected for the entire experimental period have provided several indications on the usefulness of applications that monitor people’s everyday lifestyles. In particular, obtaining feedback on the user’s adherence to the Mediterranean diet allowed us to gain an immediate awareness of the type of food consumed during meals and how this can lead to healthy eating or poor eating habits.

For some individuals, the trial led to excellent results. The following is the testimony of a doctor, Dr. Sofia Miceli: *“I take this opportunity once again to congratulate you on the great success of the App. We doctors often repeat and repeat a thousand times sentences on dietary advice and/or food and/or physical activity ... But we often have a poor response. A weight loss of even 5 kg in two months is no small thing, especially if it is also associated with a state of psychological well-being. Probably the patient understands that he must be the master of his own weight loss and the modifications of his lifestyle ... And this way he assumes all the ’responsibility’ and has for this reason an extra motivation to follow the medical advice. For me as a doctor this is what is important: he has gained motivation and an awareness of the important objectives in the field of cardiovascular illness prevention.”*

The statistical results show that out of a final sample of 24 overweight users of the Wellness App, we have obtained an improvement of the Wellness Index in 96% of cases. We can, therefore, conclude that for the participants there was a substantial improvement in their awareness about their dietary habits, as shown in the data for the Mediterranean Adequacy Index for which we have obtained an improvement for 92% of the participants. Additionally, for physical activity, the results show that in 88% of cases we have obtained an improvement of the Target Calorie Index. Less impressive results, instead, for the quality of sleep and the time spent on physical activity indexes, for which we have obtained an improvement only in 58% and 63% of the cases, respectively. It is important to note that, even if the improvement has been observed only in those latter percentages, for both indexes (the PSQI and TPA) in 33% of cases the values have remained constant, probably due to the relatively short time of the trial.

Moreover, the results indicated by the users have led to new insights aimed at improving the interaction with the app, so that it is quicker and less intrusive in the lives of users. One of these improvements could be to make the app adaptive in relation to the meal data inserted; for example, it might be possible to suggest meals in response to the weekly data on the frequency of foods consumed and in accordance with seasonal availability. The resulting 77.16 score of the SUS was good. In fact, generally, an acceptable cut-off score for such an instrument is 68, as indicated in [38,39]. Any SUS score above this cut-off is considered greater than average. Additionally, by offering a comments section after the test questions, we have collected several suggestions in order to improve the usability and navigability of the app.

Finally, we are still working on the development of new functionalities of the app in order to solve the most important open issues recorded 2. In detail, concerning the interoperability, we are now involved in a new project, “eHealthNet” (PON03PE_00128_1), and we are working to improve the app by integrating the most widespread wearable sensors, whether compliant or not to the Continua Health Alliance guidelines [40].

## Figures and Tables

**Table 1:** Behavior change techniques (BCTs) in Wellness and Health Apps.

App	Store	BCT Score
1UpFit	iTunes	5
20/20 LifeStyles Online	iTunes	4
Activious	iTunes	2
All-in Fitness: 1000 Exercises, Workouts & Calorie Counter	iTunes	5
Be Fit, Stay Fit Challenge	Google Play	5
Big Welsh Walking Challenge	iTunes	8
Croi HeartWise	iTunes	4
CrossFitr	Google Play	3
Endomondo Sports Tracker	Google Play	5
Everywhere Run!—GPS Run Walk	Google Play	5
Exercise Reminder HD Lite	iTunes	4
Faster	iTunes	4
Fit Friendzy	iTunes	5
Fitbit Activity Tracker	iTunes/Google Play	4
fitChallenge	iTunes	6
FitCoach—powered by Lucozade Sport	iTunes	6
FitCommit—Fitness Tracker and Timer	iTunes	5
Fitness War	iTunes	6
Fitocracy—Fitness Social Network, Turn Working Out	iTunes/Google Play	5
FitRabbit	iTunes	4
FitTrack	Google Play	3
Forty	iTunes	3
Get Active!	iTunes	4
Get In Gear	iTunes/Google Play	4
Go-go	iTunes	4
GymPush	iTunes	7
Healthy Heroes	iTunes	5
HIIT Interval Training TimerAD	Google Play	3
Hiking Log	iTunes	3
Hubbub Health	iTunes	7
IDoMove Work out and Win	iTunes/Google Play	4
Improver	iTunes	5
Macaw	iTunes/Google Play	5
Make your move	iTunes	5
Mobile Adventure Walks	iTunes	3
My Pocket Coach	iTunes	7
Nexercise = fun weight loss	iTunes/Google Play	5
Nike + Running	Google Play	5
Noom CardioTrainer	Google Play	5
Poworkout Trim & Tone	Google Play	4
Run Tracker Pro—TrainingPeaks	iTunes	3
RunKeeper—GPS Track Run Walk	Google Play	8
Running Club	iTunes	6
Running Log! PRO	iTunes	3
ShelbyFit	iTunes	5
Sixpack—Personal Trainer	iTunes	7
SmartExercise	Google Play	4
SoFit	Google Play	5
Softrace	Google Play	3
Strava Cycling	Google Play	5
Sworkit Pro	Google Play	6
Take a Walk Lite	iTunes	6
Teemo: the fitness adventure game!	iTunes	7
Track & Field REALTIMERUN (GPS)	iTunes	6
Tribesports	Google Play	5
Walk ’n Play	iTunes	5
Withings	iTunes/Google Play	6
**Proposed Wellness App**	**soon on Google Play**	**9**

**Table 2:** Wellness App: Trial Protocol.

Section	Specifications
**Administrative**	
Title	Wellness App Trial Protocol
Protocol version	Version 2.0
Funding	Project Pon04a2_C - MIUR D.D. 626Ric e 703Ric - Smart Health 2.0
Roles and responsibilities	The coordination of the experiment is entrusted to the University of Catanzaro. They should therefore appoint and register the names and roles of those who will join the team for the experimentation, for the stages of validation and data management, and for all the activities necessary for the performance of the entire trial. For privacy reasons, the names of the responsible managers cannot be listed here.
**Introduction**	
Objectives	The outcomes are fundamental for the trial process and the interpretation of the results. The outcomes are referred to as primary and secondary according to their relevance The primary outcome of the trial process is to determine the effectiveness of the realized Wellness App, evaluating it in relation to the Wellness Index that indicates if the app is able to stimulate the subjects to change their unhealthy behaviors, improving their diet, level of physical activity, and the conditions that affect their quality of sleep. Secondary outcome measures are: ● the usability of the app; and ● the navigability of the app;
**Methods**	
Study setting	Four districts of the Calabria Region (Crotone, Cosenza, San Giovanni in Fiore and Lamezia Terme) have been selected to be the reference point for the conducting of the trial.
Eligibility criteria	The subjects to be considered suitable for the trial process must meet the following inclusion criteria: ● Italian adults aged between 18 and 65 years; ● Subjects able to comply with the study program and the other requirements of the Protocol.
	The exclusion criteria listed below mean that the individual is ineligible for the trial: ● People aged under 18 and over 65; ● Subjects with an overt disease which include the risk factors analyzed, such as diabetes, or a cardiovascular or liver disease; ● People with neurological disorders.
Interventions	The procedures to be performed are divided into the following phases: ● Recruitment of subjects: promotion of the testing program in order to recruit the subjects; ● Information & Registration: explanation of the aims of the study. After this meeting the interested subjects receive the documents to be signed (the information, informed consent form and medical history reported in the appendix) to be delivered to the staff in charge; ● Conduct of the Trial: - First Meeting: during this phase, the investigators install the Wellness App on the mobile devices and explain its functionalities. In particular, each participant is invited to complete the Pittsburgh Sleep Quality Index (PSQI) questionnaire and the Anamnestic form. From this moment, the participant, when he/she wants, can insert the type and amount of food consumed by using the friendly interface of the app, and can upload information on the quality of the previous night’s sleep in the PSQI questionnaire. Additionally, the participant can insert information about any physical activity performed during the day, manually or automatically by wearing a sensor, such as for example the Zephyr BioHarness. It is important to note that the Android operating system is required for the installation and use of the app. For each participant who does not have an Android-based smartphone, a Mediacomm Phonepad G501 smartphone will be provided on loan for use throughout the duration of the study. - Second Meeting: during this event, the participants are updated on the progress of the study, as well as provided with a new improved version of the Wellness App realized in accordance with the indications suggested by the participants during the previous meeting. - Third Meeting: during this meeting, all the data from the mobile devices of the participants are collected, and each participant is invited to complete the PSQI questionnaire. Additionally, the investigators talk with all participants encouraging them to discuss their use of the application, any difficulties encountered during the trial, the perceived quality of the information provided, and any preferences for different features. In this latter meeting the usability test questionnaires are distributed, which the participants are requested to answer in a very spontaneous way. All the questionnaires are anonymous. During the course of the testing, the investigator should not interfere when the participants are using the application, any intervention being limited to a minimum, so as not to influence the behavior of the participant. The responsibility of the investigator is purely to reassure the participant in case of difficulty, urging her/him to continue with confidence, without ever suggesting actions to be performed and without providing explanations. Instead, she/he should, when necessary, remind the participant to comment aloud when performing actions: indicating what she/he proposes to do, what she/he sees on the screen,what she/he feels about the experience, what difficulties she/he is encountering, which doubts she/he has, etc. The investigator should note the comments made, recording the situations in which the subject expresses uncertainty or makes mistakes, or other possible problems. The investigator has the task of interrupting the subject in the event that, during the performance of the testing, she/he encounters difficulties which cannot be overcome.
Recruitment	The recruitment of the participants should be carried out by publicizing the trial process through information campaigns, using tools such as posters, journals, magazines, newspapers, the web, leaflets, brochures and meetings with project staff who will promote the aims of the study. In the recruitment phase they will outline the objectives of the trial process to the subjects, presenting to those who wish to participate the necessary paperwork, namely the information sheet, informed consent and the medical history form. Those who meet the criteria for inclusion will be invited to participate in the study.
Data collection methods and management	All data collected during the testing phase and all information required by the Protocol, in respect of each participant in the trial process must be properly registered in a clear, accurate and complete. The data collected must be signed and dated by the person who recorded them. Such data must be stored anonymously and the individual must be uniquely identified by a code. The investigator is responsible for preserving the original data of the participant. To promote the conservation of the data, the participants themselves will be involved in the process, updating their records from time to time which details about the process and about subsequent developments in the application, using their contact details (phone, address e-mail) disclosed during the “Information” phase. The data to be collected for participants who abandon the study are as follows: ● Identification of the subject; ● Cause of the abandonment of the process; ● Results obtained in the study until the time of the abandonment.
Statistical methods for the analysis of primary and secondary outcomes	For the validation, the index values calculated by the participants before and after the trial will be compared using statistical techniques to assess whether the difference between the averages is significant; in particular, for each of the four indicators, using the *t*-Student distribution method, the relative values will be calculated to determine the significance level (with a maximum threshold equal to 5%) to understand if the differences between the averages are attributable to a null hypothesis, or, on the contrary, if the hypothesis concerning the effectiveness of the application in improving that specific indicator of wellness seem to be justified. The same type of statistical calculation will be made for the global indicator IW, which is a datum derived from the other indexes. A significant improvement in the Index of Wellness confirms the success of the application. In any case, if the results for some of the indicators are not satisfactory, we will try to understand the causes by analyzing the answers given by the participants to the Usability Tests, to understand what difficulties they had encountered during the trial. From this feedback it will be possible to obtain useful information for the further improvement of the application.
Problems	In the event that the study is abandoned, the following guidelines should be implemented, depending on the cause of abandonment: ● Abandonment due to difficulties in recruiting participants: - Recommencing promotion at selected new exercise facilities selected; and - Recruitment of new participants needed for the testing; or - Ending of the trial process. ● Abandonment due to incorrect execution of the testing phase: - The investigator or the participant recommences the procedure to be followed in the testing phase; or - Ending of the trial process. Adverse events that may affect the trial process in place must be recorded in certain modules in which the following information must be specified: ● Date and time of the event; ● Stage of the process where the adverse event occurred; ● Cause of the adverse event; ● In cases where the adverse event involves a participant of the process, the data of the subject should be indicated. The investigator is responsible for assessing the severity of an adverse event and for making the subsequent decision to abandon or proceed with the study.
**Ethics and Dissemination**	
Research ethics approval	The protocol, the recruitment materials and other materials are required to be approved by the sponsors of the study and the test manager.
Changes	Any change to the protocol that can impact on the conduct of the study, compromising the safety of the participants, including changes in the objectives of the study, or significant administrative issues, require a formal amendment to the protocol. This amendment must be approved by the sponsors of the study and the test manager.
Consent or assent	Each person participating in the trial process must be fully informed about the purposes of the study, on the methods of how to conduct it, on the benefits and possible risks arising from participation in research and the ability to stop participating in the trial, at any time and without any consequences. This operation is essential because the understanding of these details and the ability to exchange information generates participation and contributes to improving the quality of the process of experimentation. All subjects are asked to sign the informed consent form and the information document, shown in the appendix, essential to ensure the protection of the confidentiality of the trial subjects, informing them about the process of testing and the treatment of personal data and the search results. In order to facilitate the task of the participants it is also desirable to provide a telephone answering service, preferably free, which is always available for further requests for clarification, comments or suggestions.
**Appendix**	
Anamnestic Form	● Personal Data: name, surname, date of birth, address, etc. ● Physiological History: smoking, alcohol consumption, etc. ● Anthropometric data: height, weight, neck size, waistline, hips (women only), etc.
Post-usability test questionnaires	Questionnaires useful for the usability evaluation. They are described in Section 6, subsection *“Usability Results”.*

**Table 3:** Participant’s Information.

Urban Area	Citizens	Installed Apps	Sheets	Usability Tests
Cosenza	10	5 (50%)	5	1
Crotone	28	21 (75%)	21	7
Lamezia Terme	17	4 (24%)	4	4
S.Giovanni in Fiore	26	10 (35%)	10	10
Total	81	40 (48%)	40	22

**Table 4:** Average values of indexes: initial and final values.

	Values at the Beginning of the Trial					Values at the End of the Trial				
Subject	IAM	TPA	PSQI	TCI	IW	IAM	TPA	PSQI	TCI	IW
Subject#1	6.28	8.27	7.62	7.28	7.33	7.54	10.00	7.62	8.77	8.58
Subject#2	6.70	10.00	6.67	8.35	8.07	8.13	10.00	7.14	9.07	8.74
Subject#3	8.24	10.00	6.67	0.52	7.28	8.57	10.00	7.14	1.00	7.55
Subject#4	5.34	0.00	5.24	2.67	3.10	6.50	2.00	6.19	4.25	4.57
Subject#5	8.24	10.00	6.67	9.12	8.71	6.35	10.00	6.19	8.17	7.84
Subject#6	5.95	10.00	4.29	7.98	7.36	7.13	10.00	4.29	8.57	7.85
Subject#7	6.77	10.00	7.14	8.39	8.18	8.45	10.00	8.10	9.22	9.04
Subject#8	6.89	0.00	7.62	3.44	4.14	8.25	2.00	7.62	5.13	5.54
Subject#9	8.13	10.00	9.00	9.06	9.05	8.48	10.00	10.00	9.24	9.37
Subject#10	4.21	10.00	7.62	6.31	7.06	6.11	10.00	7.62	3.53	7.23
Subject#11	6.17	4.00	8.10	5.08	5.59	6.41	10.00	7.62	8.20	8.11
Subject#12	6.02	4.00	8.10	0.00	4.69	6.47	10.00	8.10	1.30	7.06
Subject#13	5.39	4.00	6.67	4.70	5.02	6.41	10.00	7.40	8.21	8.07
Subject#14	6.22	10.00	6.67	8.11	7.87	7.33	10.00	6.67	8.67	8.33
Subject#15	6.00	6.00	8.50	6.00	6.42	6.50	7.00	9.00	6.75	7.13
Subject#16	8.47	5.00	5.24	6.73	6.48	7.67	7.00	6.19	7.34	7.15
Subject#17	6.73	8.00	9.00	7.36	7.64	8.95	6.80	9.00	7.87	8.06
Subject#18	7.45	3.00	1.00	5.23	4.52	8.77	3.50	2.00	6.13	5.44
Subject#19	7.54	8.00	6.67	3.76	6.92	8.46	10.00	7.14	3.76	7.97
Subject#20	6.67	2.00	5.71	4.33	4.56	7.60	3.00	6.19	5.30	5.45
Subject#21	5.68	4.00	6.19	4.84	5.06	9.29	7.00	7.14	8.14	7.98
Subject#22	7.64	8.00	6.67	7.82	7.63	8.30	10.00	6.67	9.15	8.74
Subject#23	7.51	4.00	4.76	4.38	5.36	7.86	8.00	8.10	8.05	7.98
Subject#24	7.75	3.00	5.24	5.38	5.35	8.39	4.00	5.70	6.20	6.11
